# Enhanced phosphatidylserine exposure and erythropoiesis in *Babesia microti*-infected mice

**DOI:** 10.3389/fmicb.2022.1083467

**Published:** 2023-01-04

**Authors:** Peng Song, Yu-Chun Cai, Mu-Xin Chen, Shao-Hong Chen, Jia-Xu Chen

**Affiliations:** ^1^National Institute of Parasitic Diseases, Chinese Center for Disease Control and Prevention, Shanghai, China; ^2^NHC Key Laboratory of Parasite and Vector Biology, Ministry of Public Health, Shanghai, China; ^3^WHO Collaborating Centre for Tropical Diseases, National Center for International Research on Tropical Diseases, Ministry of Science and Technology, Shanghai, China; ^4^Hainan Tropical Diseases Research Center (Chinese Center for Tropical Diseases Research, Hainan), Haikou, Hainan, China

**Keywords:** *Babesia microti*, babesiosis, erythrocyte, eryptosis, erythropoiesis

## Abstract

**Introduction:**

*Babesia microti (B. microti)* is the dominant species responsible for human babesiosis, which is associated with severe hemolytic anemia and splenomegaly because it infects mammalian erythrocytes. The actual prevalence of *B. microti* is thought to have been substantially underestimated.

**Methods:**

In this study, Bagg’s albino/c (BALB/c) mice were intraperitoneally injected with *B. microti*-infected erythrocytes, and parasitemia was subsequently measured by calculating the proportion of infected erythrocytes. The ultrastructure of infected erythrocytes was observed using scanning and transmission electron microscopes. Quantifying phosphatidylserine (PS) exposure, oxidative stress, intracellular Ca^2+^, and erythropoiesis of erythrocytes were done using flow cytometry. The physiological indicators were analyzed using a Mindray BC-5000 Vet automatic hematology analyzer.

**Results:**

Of note, 40.7 ± 5.9% of erythrocytes changed their structure and shrunk in the *B. microti*-infected group. The percentage of annexin V-positive erythrocytes and the levels of reactive oxygen species (ROS) in the erythrocytes were higher in the *B. microti*-infected group than in the control group at 10 dpi. Significant splenomegaly and severe anemia were also observed following *B. microti* infection. The parasitemia level in the *B. microti*-infected splenectomized group was higher than that of the *B. microti*-infected sham group. The population of early erythroblasts increased, and the late erythroblasts decreased in both the bone marrow and spleen tissues of the *B. microti*-infected group at 10 dpi.

**Discussion:**

PS exposure and elevated ROS activities were hallmarks of eryptosis in the *B. microti*-infected group. This study revealed for the first time that *B. microti* could also induce eryptosis. At the higher parasitemia phase, the occurrence of severe anemia and significant changes in the abundance of erythroblasts in *B. microti*-infected mice group were established. The spleen plays a critical protective role in controlling *B. microti* infection and preventing anemia. *B. microti* infection could cause a massive loss of late erythroblasts and induce erythropoiesis.

## 1. Introduction

*Babesia microti* is a tick-transmitted protozoan hemoparasite and a primary etiological agent of human babesiosis globally, thus making it a serious public health concern (Westblade et al., 2017). It has a global distribution and numerous wild and domestic animals may serve as infection reservoirs (Laha et al., 2015). It is endemic in some USA states but rare and more life-threatening in Europe (Meliani et al., 2006). The actual prevalence of *B. microti* is thought to be substantially underestimated given the typically asymptomatic nature of the infection (Xia et al., 2019). *B. microti* causes malaria-like symptoms and splenomegaly in both infected mice and humans (Djokic et al., 2018; Matsushita et al., 2021). In most cases, mild to moderate babesiosis does not require clinical admission. However, severe disease, which is associated with high risks of organ dysfunctions such as acute respiratory distress syndrome, congestive heart failure, renal failure and liver failure requires immediate attention, particularly in immunocompromised patients (Smith et al., 2020; Krause et al., 2021). Confirmed case definitions have been highly characterized by fever and anemia (Stein et al., 2017). In humans, the overall mortality rate for babesiosis is ~6%–9%, but this rises to 20% in immunodeficient patients (Onyiche et al., 2021).

*B. microti* infects mammalian erythrocytes. Although erythrocytes lack nuclei and chromosomes, they are significant health indicators during systemic or chronic inflammation because the hematological system is always exposed to peripheral inflammatory mediators and erythrocytes interact with several inflammatory molecules and compounds (Onyiche et al., 2021). Erythrocytes are biconcave discoid cup-shaped stomatocytes or spiculated echinocyte shaped under certain circumstances (Mesarec et al., 2019). The morphology of erythrocytes can be a useful tool to assess the body’s physiological state and for definitive diagnosis (Ford, 2013). Primarily, *B. microti* invades mature erythrocytes in mice. During invasion, *Babesia* secrete numerous proteins to support their development and to modify erythrocytes (Hakimi et al., 2022). Adhesive properties and permeability of infected erythrocytes are altered while cell volumes are increased (Park et al., 2015).

Erythrocytes have unique asymmetric cell membranes, and different phospholipid-based molecules are located both inside and outside of the cell (De Rosa et al., 2007). Neutral phospholipids, including phosphatidylcholine and sphingomyelin, are always located in the outer leaflet of the bilayer, whereas phosphatidylserine (PS) and anionic phosphatidylethanolamine are normally distributed in the inner monolayer. During inflammation and other conditions, PS is externalized and erythrocytes can undergo programmed death under stress *via* a process called “eryptosis” (Pretorius et al., 2016). Certain diseases, such as malaria, acute cardiac failure, lung cancer, and hemolytic anemia, have been associated with eryptosis (Lang and Qadri, 2012). Similar to the apoptosis of nucleated cells, eryptosis typically leads to cell shrinkage, cell membrane blebbing, and cell membrane scrambling with PS translocation to the erythrocyte surface (Maguire et al., 1991; Brand et al., 2003). Expression of PS on the outer membrane induces phagocytosis of damaged erythrocytes and the adhesion of erythrocytes to the endothelium (Pankova-Kholmyansky et al., 2003). Eryptosis is a form of suicidal erythrocyte death because it prevents erythrocytes from undergoing haemolysis, causing erythrocyte cell death (Qadri et al., 2017).

The spleen performs numerous immune functions and participates in hematopoiesis, blood-borne pathogen clearance, and erythrocyte homeostasis (Xue et al., 2021). Macrophages in the spleen serve mostly to filter blood and phagocytose aging red blood cells (RBC). When PS is externalized on the membrane surface, it can serve as an “eat-me” signal and be recognized by macrophages (Lemke, 2019). In recent years, more work has been done to clarify the relationship between eryptosis and *Plasmodium*. Notably, manipulating eryptosis of erythrocytes is considered to be a potential approach for malaria control (Lang et al., 2015; Boulet et al., 2018). However, whether *B. microti* can induce eryptosis, and the function of the spleen in this process, remains unknown. Herein, we used scanning electron microscope (SEM) and transmission electron microscope (TEM) technologies to observe the morphological changes of erythrocytes and flow cytometry to determine the effect of *B. microti* on erythrocytes.

## 2. Materials and methods

### 2.1. Parasites

*Babesia microti* strain ATCC^®^PRA-99TM used in this study was obtained from the Institute of Laboratory Animal Sciences, Chinese Academy of Medical Sciences (CAMS). This strain was maintained through serial passage in i.p. infected Bagg’s albino/c (BALB/c) mice. Infection was confirmed by observing for the presence of parasites in thin blood smears using an optical microscope at 3 or 5 dpi.

### 2.2. Murine infection model and parasitemia

Female specific-pathogen-free laboratory-bred house mice (BALB/c), aged 6–8 weeks, were purchased from the Shanghai Laboratory Animal Center (China). The housing and maintenance of the rodents complied with national regulations. Orbital blood samples were collected from the infected mice and diluted with sterile saline to 5 × 10^7^ *B. microti*-infected erythrocytes per milliliter. The mice in infected groups were intraperitoneally injected with 10^7^ infected erythrocytes in a volume of 0.2 ml. To ensure all mice received equal numbers of viable parasites, they were infected with equal parasite inoculum at the same time. Blood for smears was collected daily from the tail snip for 30 days post-infection (dpi) to evaluate the parasitemia level. The proportion of infected erythrocytes was calculated as previously described (Skariah et al., 2017), based on the number of infected erythrocytes per 1,000 erythrocytes. The counting was performed using a light microscope (Olympus CX41, Tokyo, Japan). The animal protocols were approved by the Laboratory Animal Welfare & Ethics Committee (LAWEC), National Institute of Parasitic Diseases of China CDC (IDP-2019-16).

### 2.3. Preparation of blood pellets for SEM and TEM

Orbital sinus blood samples were collected in ethylenediaminetetraacetic acid at 10 dpi. The blood was immediately fixed for 2 h by electron microscopy fixative at room temperature. The preservation and transportation temperature was 4°C. Each sample was washed three times for 15 min with 0.1 M phosphate buffer (PB) (pH 7.4) and stained for 1–2 h at room temperature using 1% osmium tetroxide in 0.1 M PB (pH 7.4). The cells were dehydrated for 15 min in serial ethanol concentrations of 50%, 70%, 80%, 90%, and 95% ethanol, twice in 100% ethanol, and finally in isoamyl acetate. The cells were dried using a critical point dryer (K850, Quorum, United Kingdom) and attached to metallic stubs using carbon stickers and sputter-coated with gold for 30 s. The cells were observed using an SEM (SU8100, HITACHI, Japan) and a TEM (HT7800, HITACHI, Japan).

### 2.4. Quantification of phosphatidylserine exposure

Mouse blood cells were washed twice using Ringer’s solution supplemented with 5 mM calcium chloride. To detect FITC annexin V-positive cells, the erythrocytes were suspended in an annexin-binding buffer (BD Pharmingen, San Diego, United States) with FITC annexin V (1:200 dilution, BD Pharmingen, San Diego, United States) and incubated for 15 min at room temperature. Well mixing was performed by pipetting. Finally, erythrocytes were diluted five times in the annexin-binding buffer before analysis in the FACSVerse flow cytometer (Beckman Coulter, CytoFlex S, United States) at an excitation wavelength of 488 nm (blue laser) and emission wavelength of 530 nm.

### 2.5. Quantification of oxidative stress

The oxidative stress level was determined using 2′, 7′-dichlorodihydrofluorescein diacetate (DCFDA; Sigma, Schnelldorf, Germany). Briefly, the erythrocytes (4 μl) were mixed in 1 ml Ringer’s solution, from which 150 μl of the cell suspension was centrifuged at 1,600 rpm for 3 min at room temperature. The collected cells were stained with DCFDA (10 μM) in Ringer’s solution at 37°C for 30 min and washed three times with 150 μl of Ringer’s solution. The DCFDA-loaded erythrocytes were then resuspended in 200 μl Ringer’s solution (125 mM sodium chloride, 5 mM potassium chloride, 1 mM magnesium sulfate, 32 mM hydroxyethyl piperazine ethane sulfonic acid, 5 mM glucose, and 1 mM calcium chloride; pH 7.4). The reactive oxygen species (ROS)-dependent fluorescence intensity was measured at an excitation wavelength of 488 nm and an emission wavelength of 530 nm.

### 2.6. Quantification of intracellular Ca^2+^

Calcium ion influx was evaluated using Fluo-3 Am (Invitrogen, Carlsbad, United States) staining. Erythrocytes were suspended in 200 μl Ringer’s solution supplemented with Fluo-3 Am (1 μM) and incubated at 37°C for 30 min. Thereafter, the RBCs were stained with Fluo-3 Am, rinsed, and resuspended in Ringer’s solution, and analyzed using a 488 nm blue laser and a 530 nm bandpass filter.

### 2.7. Murine splenectomy and routine blood testing

Mice were anesthetized using ether and a small incision was made under the left costal margin before ligating the splenic vessels. For mice in the sham group, the spleens were exposed but not removed. Blood was collected from the orbital sinus into a microhematocrit tube every 5 days until 20 dpi. The blood was analyzed using a Vet automatic hematology analyzer (BC-5000, Mindray, China) for animals. The physiological indicators measured included the RBC count, hemoglobin concentration (HGB), red cell distribution width standard deviation (RDW-SD), and red cell distribution width coefficient of variation (RDW-CV).

### 2.8. Erythropoiesis analysis

Erythropoiesis was analyzed as previously described (Wang et al., 2010). The mouse bone marrow cells were prepared as described previously (Yáñez and Goodridge, 2018). Briefly, the femurs of the mice were dissected from the legs, and the marrow cavity was opened. Bone marrow was harvested with cold, sterile saline until the bones appeared white. The mouse spleens were mechanically dissociated into single-cell suspensions and incubated with FITC Rat Anti-Mouse CD71 antibody (1:200, CD71-FITC, BD Biosciences, San Diego, United States) and APC Rat Anti-Mouse TER-119 antibody (1:200, TER119-APC, BD Biosciences, San Diego, United States) for 45 min. Then, cells were washed by adding 3 ml of staining buffer to each sample tube. Samples were analyzed in the Fortessa X20 flow cytometer (BD Biosciences, San Jose, CA) at an excitation wavelength of 488 nm or 640 nm and an emission wavelength of 520 nm or 680 nm.

### 2.9. Statistical analysis

Data were analyzed using Microsoft Excel^®^ software (version 2019) and GraphPad Prism (version 8.3.0). Continuous data were expressed as the mean ± SD. The difference between groups was analyzed using the student’s *t*-test, whereas analysis of variance (ANOVA) was used for multiple groups. Statistical significance was set at *p* < 0.05.

## 3. Results

### 3.1. *Babesia microti* infection induced morphological changes in erythrocytes

**Figure 1:**
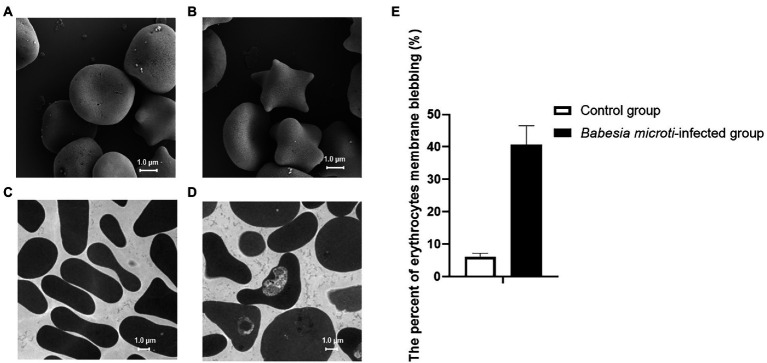
Erythrocyte morphology as seen under a scanning electron microscope (SEM) and transmission electron microscope (TEM). **(A)** SEM image showing biconcave-shaped erythrocytes of mice in the control group. **(B)** SEM image showing membrane blebbed erythrocytes of mice in the *Babesia microti(B. microti)*-infected group. **(C)** TEM image showing regularly shaped erythrocytes of mice in the control group. **(D)** TEM image showing the changed shape of erythrocytes of mice in the *B. microti*-infected group. The scale bar is 1.0  μm. **(E)** Bar graph showing the arithmetic means ± SD (*n* = 5) of the proportion of erythrocytes with a blebbing membrane without (white bar) and with (black bars) *B. microti* infection.

SEM and TEM analysis revealed that the control group had bowl-shaped erythrocytes (Figures 1A,C). In contrast, the *B. microti*-infected group had a significant increase in spurred erythrocytes that were irregularly distributed, variably sized, and with pointy projections off their surfaces (Figure 1B). *Babesia microti* were visible near the membrane of polymorphic erythrocytes under TEM (Figure 1D). Notably, 40.7 ± 5.9% of erythrocytes in the *B. microti*-infected group lost their biconcave structures, shrunk, and exhibited membrane blebbing. This proportion was significantly higher than that in the control group (*t* = 12.9, *p* < 0.01; Figure 1E).

### 3.2. *Babesia microti* infection enhanced eryptosis

**Figure 2:**
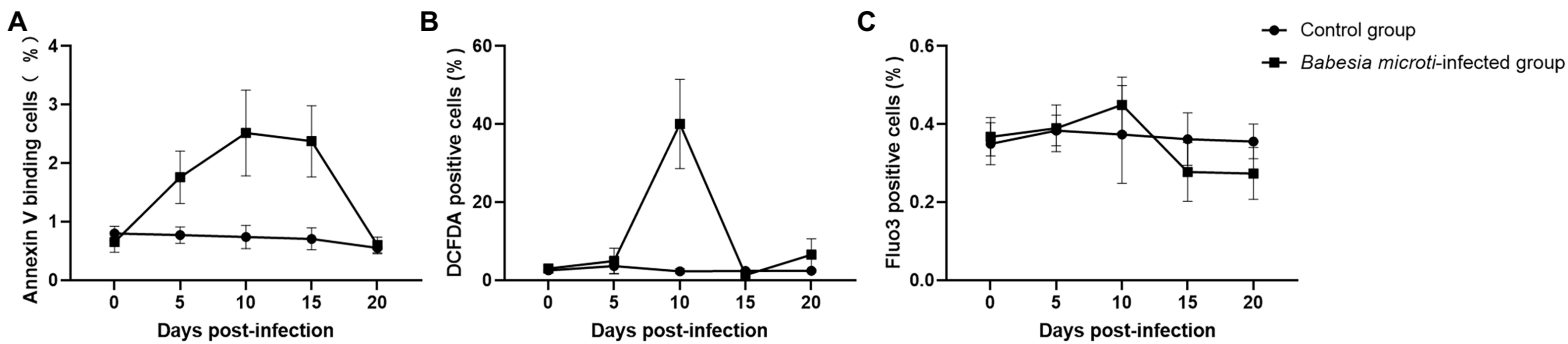
Eryptosis assays after *B. microti* infection. Line graphs showing that **(A)** *B. microti* induced more PS exposure to the cell surface of erythrocytes, **(B)** ROS levels in the erythrocytes were higher in the *B. microti* -infected group at 10 dpi, and **(C)** Calcium ion activity of erythrocytes increased slightly in *B. microti*-infected group at 10 dpi, but not significantly. The results are expressed as the mean percentage of positive cells ± the standard deviation (SD) from five mice.

We analyzed the PS surface expression, ROS level, and calcium ion activity of erythrocytes to evaluate the extent of accelerated eryptosis. PS surface expression was quantified using FACS analysis after staining the cells with fluorescein isothiocyanate (FITC)-labeled Annexin V. Of note, the percentage of annexin V-positive erythrocytes was significantly higher in the *B. microti*-infected group than in the control group at 5, 10, and 15 dpi, indicating increased cell surface expression of PS (*t* = 4.7, 5.2, 5.9, *p* < 0.01; Figure 2A). The levels of ROS in the erythrocytes were also higher in the *B. microti*-infected group than in the control group at 10 dpi (*t* = 7.4, *p* < 0.01; Figure 2B). The calcium ion activity of the erythrocytes from each group was quantified using Fluo-3 fluorescence. The calcium ion activity increased slightly in *B. microti*-infected mice than in mice in the control group at 10 dpi, but not significantly (Figure 2C).

### 3.3. *Babesia microti* infection caused splenomegaly and severe anemia

**Figure 3:**
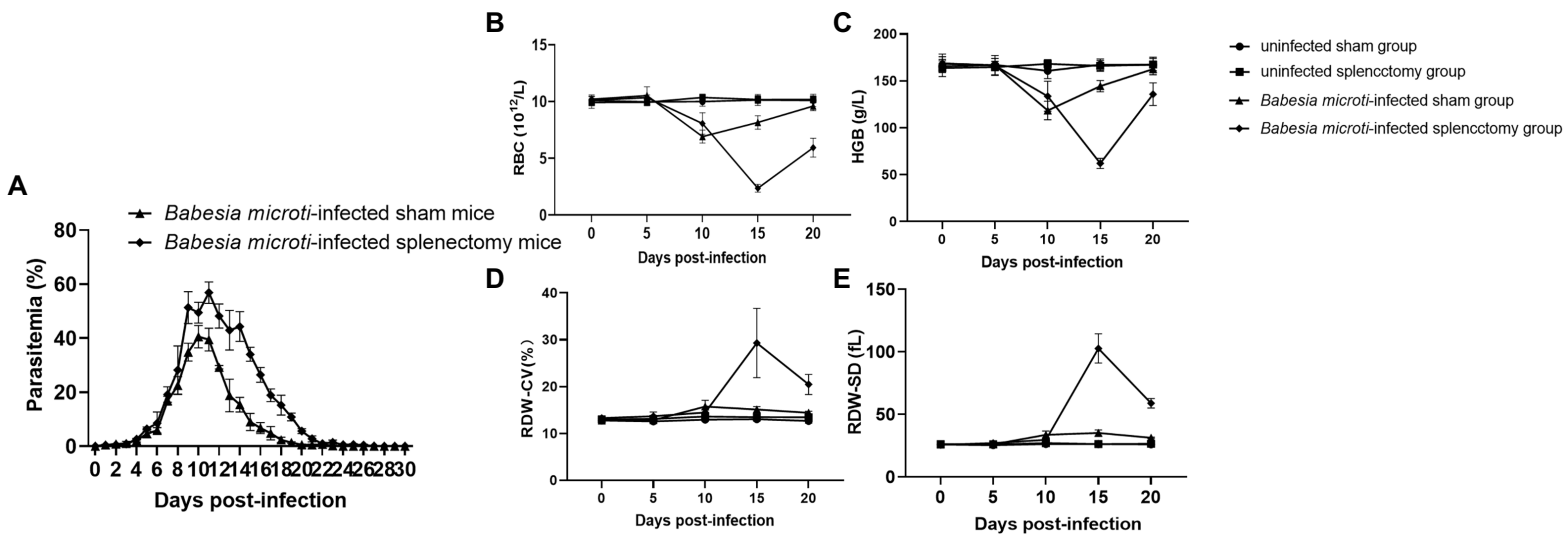
Changes in blood parasitemia and parameters after *B. microti* infection. Line graphs showing **(A)** the peak parasitemia level of the *B. microti*-infected splenectomized group was higher than that of the *B. microti*-infected sham group and persisted for ~6 days, **(B)** the red blood cell (RBC) count, **(C)** Hemoglobin concentration (HGB), **(D)** Red cell distribution width coefficient of variation (RDW-CV), and **(E)** Red cell distribution width standard deviation (RDW-SD). The results are expressed as the mean ± standard deviation (SD) of five mice.

The weights and lengths of the spleens were measured at 0, 5, 10, 15, and 20 dpi. The average spleen weights of mice in the *B. microti*-infected group were 2.34 ± 0.09, 3.36 ± 0.13, 3.08 ± 0.08, and 2.74 ± 0.25 g while those of the control group were 1.77 ± 0.16, 1.72 ± 0.08, 1.70 ± 0.14, and 1.78 ± 0.08 g at 5, 10, 15, and 20 dpi (*t* = 6.81, 23.19, 18.78, and 8.11, respectively; *p* < 0.01) (Supplementary Figure S1A). Notably, the spleen enlarged from 1.73 ± 0.05 cm at 0 dpi to 3.36 ± 0.13 cm at 10 dpi, and then recovered to 2.74 ± 0.25 cm at 20 dpi upon *B. microti* infection (Supplementary Figures S1B,C). We harvested the spleen of mice and measured the parasitemia levels in peripheral blood samples from the *B. microti*-infected sham and splenectomized groups (sham mice had the spleen while splenectomized mice had their spleens harvested) to assess the role of the spleen during *B. microti* infection. Parasitemia in both groups increased rapidly at 6 dpi and peaked at 10 or 11 dpi. Of note, 40.56 ± 4.14% of the erythrocytes from the *B. microti*-infected sham mice and 56.88 ± 3.97% of the erythrocytes from the *B. microti*-infected splenectomized mice were infected at 10 and 11 dpi, respectively. The peak parasitemia level of the *B. microti*-infected splenectomized group was higher than that of the *B. microti*-infected sham group and persisted for ~6 days. The difference in parasitemia level between the *B. microti*-infected sham and splenectomized groups at 11–20 dpi was statistically significant (*p* < 0.01). In the *B. microti*-infected sham and splenectomized groups, the parasites could not be detected in erythrocytes by 23 and 27 dpi, respectively. These results indicate that the spleen plays a defensive role in *B. microti* infection (Figure 3A).

The hematological parameters of mice in the experimental groups revealed significantly low RBC and HGB in the *B. microti*-infected sham and splenectomized mice compared to mice in the uninfected sham and splenectomized groups at 10 and 15 dpi. However, the RBCs and HGB of mice in the *B. microti*-infected splenectomized group were lower than those in the *B. microti*-infected sham group at 15 and 20 dpi (Figures 3B,C). Conversely, the RDW-CV and RDW-SD of mice in the *B. microti*-infected sham and the splenectomized group were higher than those of mice in the uninfected sham and splenectomized group at 10 and 15 dpi. However, the RDW-CV and RDW-SD of the mice in the *B. microti*-infected splenectomized group were higher than those of mice in the *B. microti*-infected sham group at 15 and 20 dpi (Figures 3D,E). Images for the morphologies of *B. microti*-infected erythrocytes are shown in Supplementary Figure S2. Multiple variabilities, including *anisochromia, acanthocytes*, and Howell–Jolly bodies, were observed in *B. microti*-infected cells.

### 3.4. *Babesia microti* infection increased erythropoiesis

**Figure 4:**
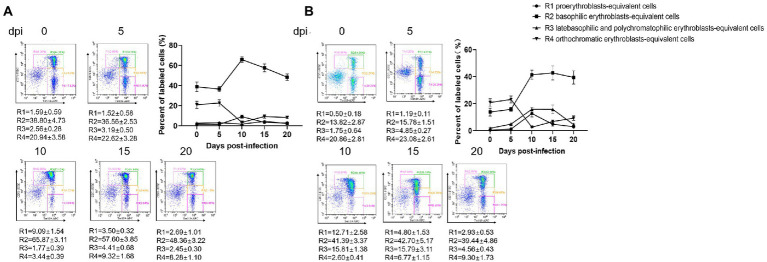
Erythropoiesis of red blood cells after infection with *B. microti*. Line graph showing the arithmetic means ± SD of the percentage of Ter119-PE and CD71-FITC labeled bone marrow **(A)** and splenocytes **(B)** isolated from mice in the *B. microti*-infected group.

Erythroid differentiation can be monitored by targeting the erythroid-specific TER119 and nonerythroid-specific CD71 antigens using flow cytometry (Koulnis et al., 2011; An and Chen, 2018). By combining Ter119 and CD71 expression, erythroid cells could be distinguished into four subpopulations: Ter119^med^CD71^high^, Ter119^high^CD71^high^ Ter119^high^CD71^med^, and Ter119^high^CD71^low^, representing the proerythroblasts-equivalent cells, basophilic erythroblasts-equivalent cells, late basophilic and polychromatophilic erythroblasts-equivalent cells, and orthochromatic erythroblasts-equivalent cells, respectively. Flow cytometry revealed a dramatic increase in the early erythroblast population (basophilic erythroblasts-equivalent cells) and a decrease in the late erythroblast population (orthochromatic erythroblasts-equivalent cells) in both the bone marrow and spleen tissues of the *B. microti*-infected group at 10 dpi (Figures 4A,B). Simultaneously, the percentages of proerythroblasts and late basophilic and polychromatophilic erythroblasts-equivalent cells were elevated in the spleen at 10 dpi (Figure 4B). The analysis of the bone marrow and spleen cells by the Wright-Giemsa staining method was not performed. These results suggest that the maturation of the late erythroblast population was significantly suppressed by *B. microti* infection and the early erythroblast population was recruited to compensate for the loss of abnormally fragile erythrocytes.

## 4. Discussion

Manipulation of human erythrocyte eryptosis is a potential approach for malaria control, however, it has not been established whether *B. microti* can induce eryptosis. In this study, we used several advanced tools, including scanning electron microscopy, transmission electron microscopy, flow cytometry, and murine splenectomy to reveal, for the first time, that *B. microti* can induce eryptosis, and clarify the roles of the spleen in controlling infection and preventing anemia.

The membranes of erythrocytes can be detrimentally affected under certain physiological or pathological conditions, causing them to undergo programmed cell death, known as eryptosis (Lang et al., 2017). Eryptosis displays some comparable hallmarks, such as cell shrinkage, membrane blebbing, and PS exposure to the cell surface, which is similar to apoptosis (Berg et al., 2001). *Babesia* spp. use gliding motility to migrate and penetrate the erythrocytes. Merozoites of Babesia can sometimes egress from erythrocytes without rupturing the membrane (Asada et al., 2012). Erythrocytes in the *B. microti*-infected group shrunk, and their cell membranes exhibited blebbing, which were typical features of eryptosis. We thus detected the degree of PS surface exposure, ROS level, and calcium ion activity in erythrocytes to confirm if *B. microti* induced eryptosis. Notably, the level of PS exposure was higher in the *B. microti*-infected group than in the control group at 5, 10, and 15 dpi, suggesting that eryptosis occurred. PS promotes blood coagulation and plays a pivotal role in recognizing and removing defective eryptosis *via* a PS-recognizing receptor on phagocytic cells (Zwaal et al., 2005). The increase in intracellular ROS and calcium entry are also important factors that promote eryptosis (Fink et al., 2019). The ROS activity was also higher in the *B. microti*-infected group than in the control group at 10 dpi. ROS are thought to play a dual role in the physiological functioning of body systems. Though they are toxic byproducts of aerobic metabolism, they are also involved in regulating signal transductions (Mittler, 2017). In malaria, ROS are generated as a byproduct of parasite hemoglobin metabolism in erythrocytes. Anti-oxidative proteins in *B. microti* have been reported as potential targets of anti-parasitic drugs (Huang et al., 2018). Ca^2+^ plays a key role in erythrocyte invasion; increased calcium concentration stimulates eryptosis (Singh et al., 2012). In this study, calcium concentration did not change significantly during *B. microti* infection, suggesting that the calcium ion transport channel in cell membranes was not activated. However, a morphological comparison of erythrocytes infected with *B. microti* and normal erythrocytes was not performed. The changes in *B. microti* infected-erythrocytes could be elucidated further using the DNA/RNA specific dyes, including Hoechst 33342 (Moles et al., 2015), Syto16 (Brand et al., 2008), and flow cytometry.

Eryptosis functions as a protective mechanism in some cases because it provides the erythrocytes with another form of erythrocyte cell death other than haemolysis (Bartolmäs et al., 2018). Homeostasis between eryptosis and antieryptosis mechanisms is vital in maintaining normal erythrocyte count in the blood, thereby preventing irregularities. Human babesiosis is usually associated with severe hemolytic anemia and splenomegaly (Dumic et al., 2020). Haemolysis of injured or damaged erythrocytes causes the release of erythrocyte contents into the bloodstream. In the same line, the spleen plays an important role in hematopoiesis and erythrocyte clearance (Lewis et al., 2019). We used a splenectomized mice model to observe the differences in parasitemia and hematological parameters between mice in *B. microti*-infected sham and splenectomized groups. Splenectomized mice models have been used to study the functions of splenic T_reg_ cells, the roles of the spleen in decreasing platelet counts, and the filtering functions of the spleen (Manning and McDonald, 1997; Grunewald et al., 2017; Wang et al., 2019). Preliminary results suggest that the integrity of splenic functions affects liver morphology and that the spleen has a protective function in autoimmune hepatitis. In this study, differences between the sham and splenectomized groups were subsequently used to evaluate the anemic condition, and the spleen function in *B. microti-infected* mice. Unlike severe combined immunodeficient (SCID) mice and nonobese diabetic SCID mice, *in vivo* models using BALB/c mice have shown that *B. microti* infection can resolve spontaneously after reaching peak parasitemia (Lu et al., 2012). Parasitemia remained higher in mice in the *B. microti*-infected splenectomized group for several days before gradually decreasing compared to mice in the *B. microti*-infected sham group. Moreover, the extent of splenomegaly was consistent with the level of parasitemia. Apart from the spleen, which is a major site for removal of infected erythrocytes, the liver and lung tissues can also exhibit severe injury as complications of *B. microti* infections (Alvarez De Leon et al., 2019; Hu et al., 2021). It has been reported that a splenectomized patient infected with babesia recovered with only symptomatic treatment (Rosner et al., 1984). This indicates that the spleen is critical, but not the only organ involved, in controlling *B. microti* infection.

The RBCs and HGB values were significantly lower in the *B. microti*-infected splenectomized group compared to the *B. microti*-infected sham group, indicating more severe anemia occurred in mice of the splenectomized group after *B. microti* infection. Therefore, the absence of a spleen may promote further damage to the erythrocytes. The increases in RDW-CV and RDW-SD at 15 dpi indicate a significant change in the morphology of the erythrocytes. Consistent with a previous report (Park et al., 2015), the cellular volume of the erythrocytes increased as a result of *B. microti* infection when observed under 3D holographic microscopy. Changes in hematological parameters reflect the deregulation of erythrocyte homeostasis and the declining capacity of the spleen to clear abnormal erythrocytes (McKenzie et al., 2018).

Anemia ensues when increased eryptosis results in the loss of circulating erythrocytes without the combined increase in erythropoiesis and sustained increase of reticulocytes (Singh et al., 2012). Erythropoiesis is defined as the generation of erythrocytes from hematopoietic stem and progenitor cells through a series of intermediate progenitors (Nandakumar et al., 2016). Erythroid burst-forming units and erythroid colony-forming units (CFU-E) are early progenitors in the erythroid lineage (Palis and Koniski, 2018). CFU-E progenitors differentiate through several morphologically defined stages which can be grouped into four populations: proerythroblasts, basophilic erythroblasts, polychromatophilic erythroblasts, and orthochromatophilic erythroblasts. Erythropoiesis results showed that the percentage of basophilic erythroblasts-equivalent cells in the bone marrow and spleen dramatically increased, and the percentage of orthochromatic erythroblasts-equivalent cells decreased, suggesting that the main cause of severe anemia in *B. microti* infection might be the loss of erythrocytes coupled with the inability of enhanced erythropoiesis to compensate for this loss fully. Since it is challenging to accurately quantify the absolute number of erythroblasts in the bone marrow or spleen, we did not determine if there were changes in the production of erythrocyte precursors.

Giemsa or Wright’s blood smear staining is a useful and convenient method for definitive babesiosis diagnosis. Macrocytic anemia was observed in the *B. microti*-infected group, and the mean corpuscular volume was higher in the infected group than in the non-infected group. However, cytopreps of single-cell suspensions of the bone marrow and spleen from *B. microti-*infected mice and cell morphology analyses using Wright-Giemsa were not performed in this study. The outcome could reveal any alteration in the ratio of erythroid to granulocytic precursors or lymphocytes (Slavova-Azmanova et al., 2013). Additionally, whether *B. microti* infection influences the lifespan of erythrocytes remains unclear. The molecular mechanism underlying *B. microti*-induced eryptosis and spleen modulations need further investigation.

## 5. Conclusion

Erythrocytes in the *B. microti*-infected group underwent eryptosis. The increased PS exposure and ROS activity in the *B. microti*-infected mice model confirmed eryptosis, an erythrocyte’s suicidal type of cell death. These results suggest that the spleen plays a protective role in controlling *B. microti* infection and preventing anemia. *Babesia microti* infection could cause a massive loss of late erythroblasts and induce erythropoiesis.

## Data availability statement

The raw data supporting the conclusions of this article will be made available by the authors, without undue reservation.

## Ethics statement

The animal study was reviewed and approved by the Laboratory Animal Welfare & Ethics Committee (LAWEC), National Institute of Parasitic Diseases of China CDC.

## Author contributions

PS and Y-CC: data curation and writing the original manuscript draft. PS, Y-CC, and M-XC: methodology. S-HC and J-XC: writing, review and project supervision. All authors contributed to the article and approved the submitted version.

## Funding

This project was funded by the Shanghai Municipal Health Commission (20194Y0046), the Shanghai Natural Science Foundation (21ZR1469900), the National Parasitic Resources Center, and the Ministry of Science and Technology (NPRC-2019-194-30).

## Conflict of interest

The authors declare that the research was conducted in the absence of any commercial or financial relationships that could be construed as a potential conflict of interest.

## Publisher’s note

All claims expressed in this article are solely those of the authors and do not necessarily represent those of their affiliated organizations, or those of the publisher, the editors and the reviewers. Any product that may be evaluated in this article, or claim that may be made by its manufacturer, is not guaranteed or endorsed by the publisher.

## Supplementary material

SUPPLEMENTARY FIGURE S1 General features changes of the spleen in non-infected and *Babesia microti*-infected mice. The weights **(A)**, lengths **(B)**, and images **(C)** of spleens in non-infected and *Babesia microti*-infected mice.

SUPPLEMENTARY FIGURE S2 Morphologies of *Babesia microti*-infected erythrocytes in blood smears. Images of erythrocytes infected with *Babesia microti* at 0dpi **(A)**, 5dpi **(B)**, 10dpi **(C)**, 15dpi **(D)**, and 20 dpi **(E)**.
