# Erratum: Enhanced phosphatidylserine exposure and erythropoiesis in *Babesia microti*-infected mice

**DOI:** 10.3389/fmicb.2023.1157549

**Published:** 2023-02-21

**Authors:** Frontiers Production Office

**Affiliations:** Frontiers Media SA, Lausanne, Switzerland

**Keywords:** *Babesia microti*, babesiosis, erythrocyte, eryptosis, erythropoiesis

Due to a production error, the DOI for this article was incorrectly registered as 10.3389/fmicb.2023.1083467. The correct DOI for the article is 10.3389/fmicb.2022.1083467.

The publisher apologizes for this mistake. The original article has been updated.

