# Supplementary figures and images for: Enhanced phosphatidylserine exposure and erythropoiesis in Babesia microti-infected mice
Source: Front Microbiol. 2023 Jan 4;13:1083467. doi: 10.3389/fmicb.2022.1083467 (PMC9846230; doi:10.3389/fmicb.2022.1083467)

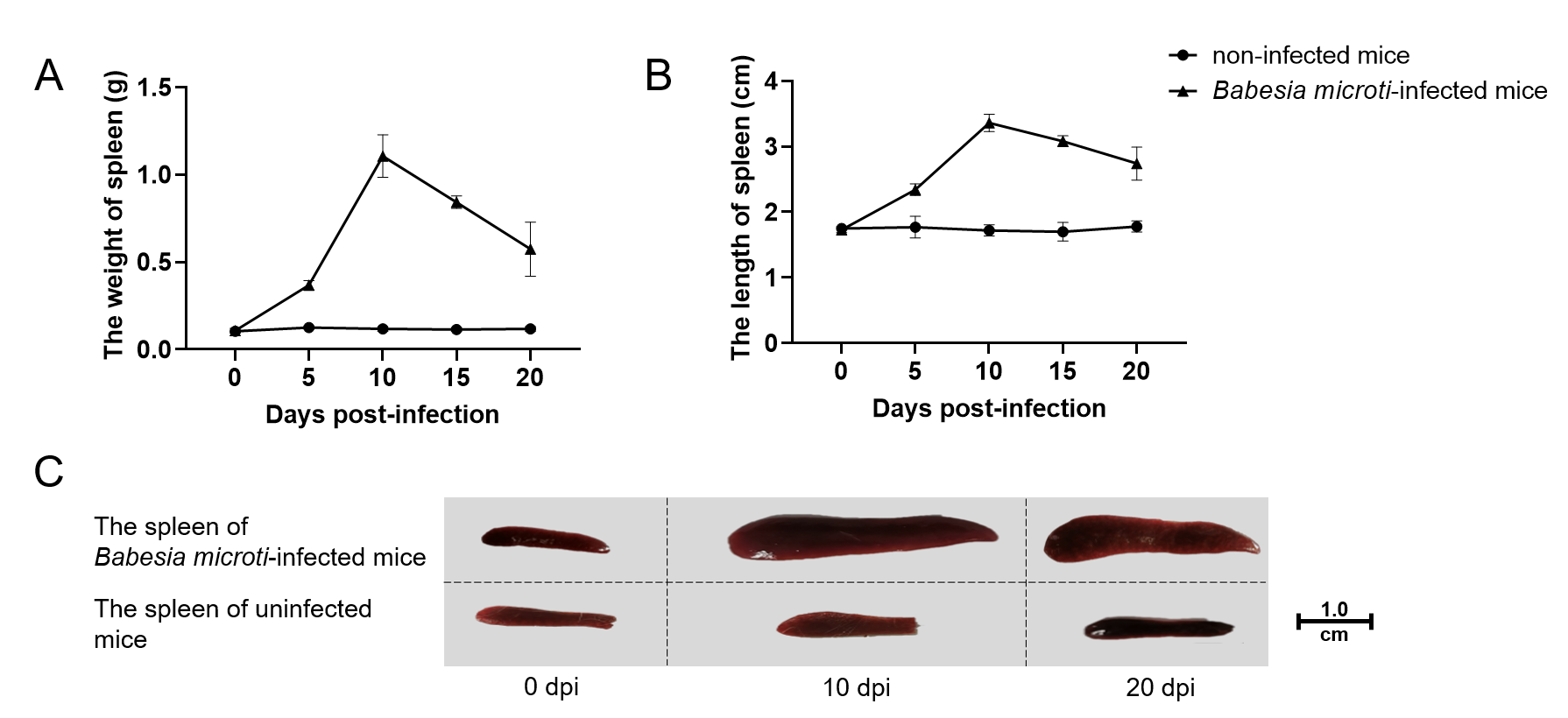

Supplement: SUPPLEMENTARY FIGURE S1 — General features changes of the spleen in non-infected and Babesia microti-infected mice. The weights (A), lengths (B), and images (C) of spleens in non-infected and Babesia microti-infected mice. [file Image_1.tif]

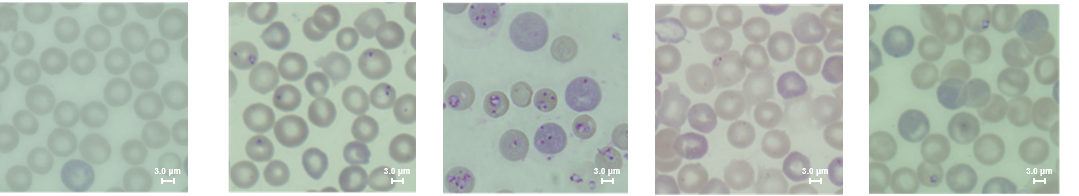

Supplement: SUPPLEMENTARY FIGURE S2 — Morphologies of Babesia microti-infected erythrocytes in blood smears. Images of erythrocytes infected with Babesia microti at 0dpi (A), 5dpi (B), 10dpi (C), 15dpi (D), and 20 dpi (E). [file Image_2.tif]
